# Supplementary material for: Developing a Sustainable Antimicrobial Stewardship (AMS) Programme in Ghana: Replicating the Scottish Triad Model of Information, Education and Quality Improvement
Source: Antibiotics (Basel). 2020 Sep 23;9(10):636. doi: 10.3390/antibiotics9100636 (PMC7598579; doi:10.3390/antibiotics9100636)
Supplement: Supplementary file 1 [file antibiotics-09-00636-s001.pdf]

Supplementary Materials

Figure S1. Antimicrobial stewardship training plan—Ghana 2019

Training detailed below to be delivered on Day 1 and repeated on Day 2 to a multi-professional group of 10-15 participants each day. On Day 3, teams will come together to agree next steps, undertake informal behaviour training and project planning

Time	Session	Content	Format
9.00–9.10	Welcome and introductions	Lead partners explain aims of the day<br>Speakers and hospital staff introduce themselves	Lecture
9.10–9.30	Antimicrobial resistance (AMR)	What is AMR and why is it important<br>WHO and Ghana action plans	Lecture
9.30–9.50	Antimicrobial stewardship (AMS)	What is AMS and how can it help us tackle AMR<br>Getting started with AMS in your hospital and adopting a multi-professional approach	Lecture
9.50–10.20	Antibiotic use data	Importance of measuring antibiotic use<br>African Perspective<br>Keta PPS results May 2019	Lecture
10.20–10.40	Common infections	What are common infections, what bacteria cause them and how should they be treated	Lecture
10.40–10.50	Antibiotic guidelines	The importance of guidelines<br>Ghana Standard Treatment Guidelines	Lecture
10.50–11.10	Everyone has a role to play	Roles in AMS :<br>Prescribers / Pharmacists/ Nurses / Lab Staff<br>Nurse focus	Lecture
11.45–12.45	Case studies	Using antibiotics in hospital practice<br>Includes group feedback for each case	Group discussion
12.45–13.15	Skills Practise	Practise interventions to challenge non-compliance with policy	All
13.15 - 13.45	Group Discussion	What can we do to improve antimicrobial stewardship in our hospital?<br>Agree main points for meeting on Thursday	discussion
13.45–13.55	Q & A	Opportunity to raise queries with speakers	Discussion
13.55–14.00	Closing remarks	Summary and reflections on the day	Lecture

Day 3: How to put some of the ideas and action plan into practice: Behaviour change and QI project planning

9.00-10.00	Whole team meeting	Feedback from teams on training session and action plan of key areas to tackle	Core Improvement Group to lead	Discussion

10.00- 10.30	Behaviour Session	COM- B model and other useful tools	Change Xchange	Interactive teaching
10.30- 12.00	Project Planning		Core Improvement Group	Discussion

Figure S2. Ghana Police Hospital AMS Action Plan—2019/2020

Short-term plans

ACTIONS	TIMESCALES
Laboratory antimicrobial susceptibility surveillance–retrospective data collection	Jan 2020
Completion of guidelines for surgical/maternity unit	Dec 2019
Cascading of training of AMS to staff	Jan 2019
Routine collection of antimicrobial prescribing at Pharmacy/Ward	Starts Dec 2019
Conduct PPS survey	10th February 2020

Mid-term–Long term plans

ACTIONS	TIMESCALES
Monthly AMS meetings	ongoing
Laboratory antimicrobial susceptibility surveillance-prospective quarterly data collection	Starts in Jan 2020
Routine collection and analysis of antimicrobial prescribing at Pharmacy/Ward	June 2020
Presentation of hospital PPS study results (2019 and interventions made) at Clinical Meeting (March 2020)	March 2020
Cascading of training of AMS to staff	Jan 2019
Share findings of PPS results with DDTC	May-June 2020
Establishment of AMS monitoring team at wards/Pharmacy	June 2020
Patients sensitization on AMR and prudent use of antimicrobials	Nov 2020
Presentation of hospital AMS and PPS results (Feb 2020) at Clinical Meeting/Hospital Management	October–November 2020
Routine collection and analysis of antimicrobial prescribing at Pharmacy/wards	December 2020
Submit analysed results of routine antimicrobial prescribing and use patterns for publication	December 2020

Figure S3. Updated Action plan Keta Hospital (Feb 2020)

Code	Action	Next Steps	Actions taken (up to Feb 2020)
**Green**	Train all prescribers and clinical staff	• Abbreviated content<br>• Identify and delegate presenter<br>• Identify opportunities<br>• List of attendees	Achieved<br>118 additional staff trained, ongoing training will continue
**Green**	Ensure all staff can access the guideline	• Access to app as part of training<br>• Encourage staff to download<br>• Summary of common infection posters<br>• Downloaded version to PCs	Achieved<br>Additional App training given<br>Posters with treatment guidelines now available in all clinical areas
**Green**	Educate Patients about using antibiotics and about the need for staff to access guidelines on their phone	• Posters<br>• Videos<br>• Infographic on how to download<br>• WHO website<br>• Other languages<br>• Leaflets with key messages for conversations	In progress<br>2x education sessions in OPD<br>Education in diabetes and Hypertension Clinic
**Green**	Promote teamwork to support AMS	• Friday meetings<br>• Socialising<br>• AMT plan	In progress<br>AMT meetings started and teams working at ward level and across hospital on AMR
**Green**	Develop process to encourage staff to check prescriptions against the guideline and query any prescription that doesn't match	• Standardisation<br>• Measurement<br>• Raise at ward rounds<br>• Feedback from using the app	In progress<br>Poster guidelines are now in all clinical areas to facilitate discussion
**Amber**	Develop surgical guidelines	• Anaesthetist<br>• Work with GPH<br>• Evidence review and discussion	Not started
**Amber**	Gaps in guidelines and local development/ adaptation of policy	• PPS data<br>• Identify gaps<br>• Guidelines team	In progress<br>2nd pps completed<br>AMT considering gaps identified by PPS: Paediatric guidelines, Obs and Gyn Surgery, Sepsis, UTI vs STI, General Surgical<br>QI project compliance with pneumonia policy in OPD
**Amber**	Spreading message of AMS via local community e.g. churches and local radio station	• Engage Reverends and identify key people in community<br>• Public health<br>• Local radio contacts	Not started
**Red**	Establish microbiology testing	• Health economist modelling cost effectiveness<br>• National solution may be most likely	Not started

Green *= short term/easy to achieve;* ***Amber*** *= medium term/more difficult;* ***Red*** *= long term/difficult*
